# The Changes in Maternal Mortality in 1000 Counties in Mid-Western China by a Government-Initiated Intervention

**DOI:** 10.1371/journal.pone.0037458

**Published:** 2012-05-21

**Authors:** Juan Liang, Xiaohong Li, Li Dai, Weiyue Zeng, Qi Li, Mingrong Li, Rong Zhou, Chunhua He, Yanping Wang, Jun Zhu

**Affiliations:** 1 National Office for Maternal and Child Health Surveillance, West China Second University Hospital, Sichuan University, Chengdu, Sichuan, China; 2 National Center for Birth Defects Monitoring, West China Second University Hospital, Sichuan University, Chengdu, Sichuan, China; 3 Obstetric and Gynecologic Department, West China Second University Hospital, Sichuan University, Chengdu, Sichuan, China; The University of Adelaide, Australia

## Abstract

**Background:**

Since 2000, the Chinese government has implemented an intervention program to reduce maternal mortality and eliminate neonatal tetanus in accordance with the Millennium Development Goals 5. To assess the effectiveness of this intervention program, we analyzed the level, trend and reasons defining the maternal mortality ratio (MMR) in the 1,000 priority counties before and after implementation of the intervention between 1999 and 2007.

**Methodology/Principal Findings:**

The data was obtained from the National Maternal and Child Health Routine Reporting System. The intervention included providing basic and emergency obstetric equipment and supplies to local medical hospitals, and also included providing professional training to local obstetric doctors, development of obstetric emergency centers and “green channel” express referral networks, reducing or waiving the cost of hospital delivery, and conducting community health education. Based on the initiation time of the intervention and the level of poverty, 1,000 counties, containing a total population of 300 million, were categorized into three groups. MMR significantly decreased by about 50%, with an average reduction rate of 9.24%, 16.06%, and 18.61% per year in the three county groups, respectively. The hospital delivery rate significantly increased. Obstetric hemorrhage was the leading cause of maternal deaths and significantly declined, with an average decrease in the MMR of 11.25%, 18.03%, and 24.90% per year, respectively. The magnitude of the MMR, the average reduction rate of the MMR, and the occurrence of the leading causes of death were closely associated with the percentage of poverty.

**Conclusions/Significance:**

The intervention program implemented by the Chinese government has significantly reduced the MMR in mid-western China, suggesting that well-targeted interventions could be an efficient strategy to reducing MMR in resource-poor areas. Reduction of the MMR not only depends on conducting proven interventions, but also relies on economic development in rural areas with a high burden of maternal death.

## Introduction

Millennium Development Goal 5 (MDG5) specifically aims to reduce worldwide maternal mortality ratio (MMR) by 75% between 1990 and 2015 [1]. However, during 1990–2008, the yearly rate of decline of the global MMR was 1.3% [2]. The rate of decline is much slower than the Millennium Development Goals 5 (MDG5) target of 5.5% decrease in global MMR per year [3]. Noticeably, maternal mortality has a high degree of inequality across the world's regions [4], [5]. In 2005, about 95% of maternal deaths worldwide occurred in two regions, sub-Saharan Africa (50%) and Asia (45%). Furthermore, about 48% of overall maternal deaths in these regions were concentrated in just five countries [5], [6]. To ensure achievement towards MDG5, the Countdown to 2015 has been initiated worldwide to track proven interventions and maternal mortality in those developing countries with a high or very high burden of maternal mortality [7], [8].

In spite of a slow reduction rate and high geographical inequality in global MMR in the past 15–20 years, exciting progress has been made in many developing countries and many efficient interventions have been proved to remarkably reduce MMR in many developing countries. Examples include strengthening control of infectious diseases in Sir Lanka, conducting of contraceptive strategies in Bangladesh, improving accessibility to in-hospital care and midwife services in Egypt, Honduras, Malaysia and Thailand, and the Maternal Mortality Reduction Strategy in Mongolia [9]–[15]. In China, in accordance with MDG5 to reduce maternal deaths, the State Council Committee for Women and Children together with the Ministry of Health and the Ministry of Finance have implemented an intervention so-called “reducing maternal mortality and eliminating neonatal tetanus” (“Reducing and Eliminating”) since 2000 [16]. Due to the high geographic inequality of the MMR in China (much higher in remote/rural areas than in coastal/urban areas) [17], this program has specifically focused on the remote and rural areas in mid-western which have a high or very high burden of maternal mortality. The program includes improving the quality of local obstetric healthcare services; developing an emergency system; reducing or waiving the cost of hospital deliveries; conducting health education; and improving supervision and management of obstetric services. It is considered the single, longest lasting project with the largest governmental funding and the broadest coverage since the People's Republic of China was founded. In this study, to assess the effectiveness of the intervention on maternal mortality, we analyzed the level and the trend of the MMR, as well as the leading causes of maternal death from 1999–2007 in priority counties before and after implementation of the intervention.

## Methods

### Selection of priority counties

There were a total of 1,000 priority counties representing approximately 300 million people in this project. The priority counties were mainly identified in under developed rural areas where the level of poverty was very high and where a majority of the nationwide maternal deaths occurred. The 1,000 priority counties were categorized into three groups based on the time they participated and the proportion of their population living in poverty. The first group (Group 1) included 378 counties across 12 provinces, autonomous regions and municipalities in mid-western China. They were the first group where the “Reducing and Eliminating” program was initiated in 2000. This group included Inner Mongolia, Jiangxi, Hunan, Chongqing, Sichuan, Guizhou, Yunnan, Tibet, Gansu, Ningxia, Qinghai and Xinjiang. The second group (Group 2) of 62 counties across four provinces (Jilin, Hubei, Guangxi and Shaanxi) including Xinjiang Production and Construction Crops, participated in 2002. In 2005, the third group (Group 3) of 560 counties participated. This group included six provinces: Hebei, Shanxi, Heilongjiang, Anhui, Henan, and Hainan. The proportion of individuals living in poverty in the three county groups was 72.62%, 53.41%, and 39.03%, respectively. The definition of poverty in China is based on individual income received annually and the poverty level set varies with time. The poverty level was set at 625 RMB of an individual's annual income in 2000, 637 RMB in 2002, and 683 RMB in 2005 [18].

### Interventions

The “Reducing and Eliminating” intervention contains six objectives specifically targeted to the under developed medical care systems in the priority counties. To accomplish each objective, there were several implemented activities and government annual funding between 2000 and 2007 (Table 1) [16]. The intervention included providing basic and emergency obstetric equipment to village-, township- and county-level medical hospitals to improve quality and accessibility of obstetric services. This equipment included delivery packages, medicine, obstetric tables, vacuum extractors, ambulances, anesthesia machines and maternal monitors. In addition the intervention provided the local obstetric doctors regular professional training with the obstetric knowledge and skills to improve quality of obstetric services in the priority counties. These skills included midwifery skills, manual removal of placenta, repair of vaginal laceration, and cesarean section. Development of obstetric emergency centers and express referral networks, called a ‘green channel’ and reducing or waiving the cost of hospital delivery to encourage rural women to go to the hospital to deliver to reduce maternal mortality risks were other aspects of the overall intervention. Finally, the intervention also included educating the community in easily acceptable methods (free brochures, media, TC, VCD, performances, and seminars) about maternal health, benefits of hospital delivery, and possible risks of traditional delivery.

**Table 1:** Major interventions of “Reducing and Eliminating” project in China, 2000–2007.

Objective	Implemented Activities	Financial sources (RMB,¥)
To improve obstetric healthcare services in county-, township- and village-level hospitals	1. Equip county-level medical centers with necessary equipment for comprehensive emergency obstetric services; 2. Equip the central township hospitals with necessary equipment for basic emergency obstetric services, and general township hospitals with common obstetric equipment; 3. Develop standards for township hospital's obstetric facilities and personnel professional skills, and establish assessment and referral system for high-risk pregnancies; 4. Develop standards for assessment of village-, township- and county-level medical centers and hospitals; 5. Provide particular professional training to doctors and medical workers at different levels of medical centers and hospitals, such as modern delivery procedures, identification of high-risk pregnancy, critical obstetric cares; 6. Send experts from province- and city-level hospitals to county-, township- and village-level medical centers and hospitals for 3–6 months to instruct and train doctors and medical workers there	Funded by the central and local government together (Central+local government): **For 2000–2001:** 100 million+100 million; **For 2002–2003:** 30 million+30 million; **For 2004:** 50 million+50 million; **For 2005:** 130 million+130 million; **For 2006:** 440 million+60 million; **For 2007:** 440 million+70 million; **Total:** 1.19 billion+0.44 billion
To establish obstetric emergency centers at county level	Develop standards for obstetric emergency centers at county level. Setup one or two centers each country and equip them with essential emergency equipment, medicines and blood products, and assemble emergency teams by recruiting experienced doctors specialized in different divisions, such as obstetrics and gynecology, internal medicine, pediatrics, and laboratory medicine. The emergency centers provide 24-hour services and referral of high-risk pregnancies and women with pregnancy in their counties	
To develop an express obstetric emergency service, so called “green channel”	Establish a referral network for high-risk pregnancies and women with pregnancy at three levels (village, township and county), mainly dependent on medical facilities in county-level hospitals. List contact information of all hospitals at three levels available for referral. Ensure ambulances are available 24-hours. Establish criteria for the referral system, and regulations on network management	
To expand hospital delivery to rural areas	Reduce or waive the cost of hospital delivery to medical centers and hospitals at all levels to encourage rural women to use hospitals for delivery. Provide proportional reimbursement for the cost of hospital delivery thought a medical care system specifically to rural areas in China, referred to as the Country Cooperative Medicare	
To encourage health education	Establish a community health education model, so- called “women as the core, the family as the best place” to conduct face-to-face education on maternal care, safe delivery, and other related health knowledge. Provide information on healthcare services available in their communities	
To strengthen supervision and guidance	Establish guidelines for supervision and guidance for obstetric healthcare services at different levels (national, provincial and county), and conduct an inspection and assessment annually in order to avoid, and timely correct any mistakes	

### Data collection

The data analyzed in this study was obtained from the National Maternal and Child Health Routine Reporting System (MCHRRS) supported by the Ministry of Health [19]. Data collection was based on the three-level administration network of the Maternal and Child Healthcare (MCH) Institutions. Trained village health workers collected information on live birth, maternal death, and prenatal and postnatal conditions. They then filled out a monthly surveillance form and submitted it to the doctors in the township health centers. The doctors then verified all the data from the different villages, filled out a surveillance summary form, and reported it to the county-level MCH Institutions every six months. The submitted data was verified by the county-level MCH and reported to the province-level MCH every year. To ensure accuracy, all the data was carefully reviewed by the MCH Institutions along with the corresponding hospitals, birth control offices, and household registration offices.

Every case of maternal death in the 1,000 priority counties was carefully investigated by professional doctors from the county-level MCH. According to WHO guidelines [20], an investigating verbal autopsy was conducted for those died at home, while for those died in hospital, an investigation was based mainly on medical records and interviews with medical personnel and family members. Once every three months, the Assessment Committee of Maternal Death conducted a review of these cases including verifying causes of the deaths, analyzing related factors, and suggesting potential interventions. An assessment report was submitted afterward to the county-level health administration department and the province-level Assessment Committee of Maternal Death. The province-level assessment committee conducted a second review on those reported cases every six months. Any contradiction in those cases was clarified by the committees at both the county and province levels. All cases of maternal deaths were defined and classified based on ICD-10 [21].

During the period of 2000–2007, a total of 17,786 cases of maternal death have been reported in the three county groups; 17,076 cases have been verified by professional doctors and the province-level Assessment Committee of Maternal Death based on ICD-10. The ascertainment rate of maternal death was 96%. 4,781 maternal deaths occurring at home have been investigated using verbal autopsies by the county-level doctors and the causes of death in 90% of the cases were consistent with those assessed by the province-level assessment committee. Data analyzed in this study have been all verified by the province-level assessment committee.

This study was granted a waiver by the Ethics Committee of West China Second University Hospital of Sichuan University.

### Statistical analysis

The baselines of maternal mortality in the three county groups were calculated based on data one year before the intervention program was implemented. The “Reducing and Eliminating” intervention mainly focuses on advocating hospital delivery to increases hospital delivery rate (HDR). Previous studies have confirmed that there is a negative correlation between HDR and MMR. In this study, the ratio of average change speed (RACS) of MMR and HDR was calculated to analyze their covariant relationship, which is the influence of increase in HDR on reduction of MMR in a specific period between year_0_ and year_t_. The average change speed (ACS) is the annual change speed in average for an indicator during a period. A positive ACS indicates an increase in the speed, and a negative one indicates a decline in the speed. The RACS reflects the efficacy of reduction in MMR by increase of HDR. The calculation formula of RACS is as follows.
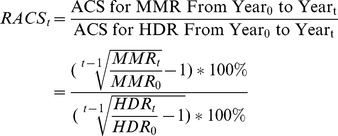



MMR and cause-specific mortality were calculated by taking the live births as the denominator and the corresponding number of deaths as the numerator. Having been considered the change of MMR in each year during a period, Poisson regression assay was used to estimate the average annual reduction rate (AARR) of MMR and cause-specific mortality ratio. SAS (version 9.0) was used to analyze the proportion of all causes in maternal death and its changing margin.

## Results

### Maternal mortality and hospital delivery rate in the studied counties during 1999–2007

**Figure 1:**
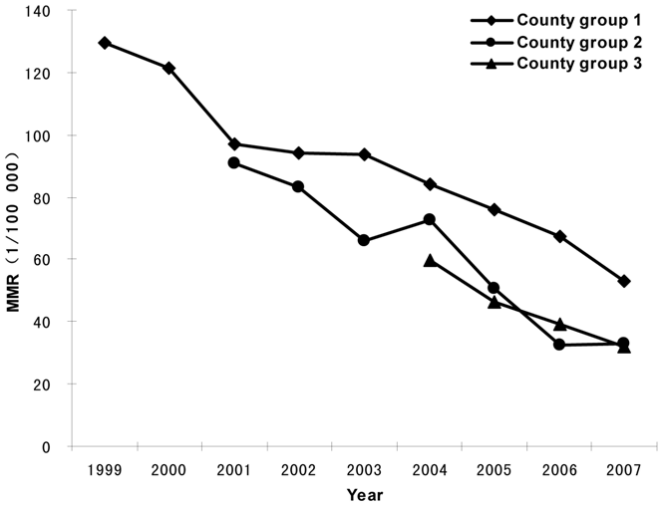
Trends of maternal mortality ratio in three county groups, 1999–2007.

MMR was the highest in the first group among the three county groups each year throughout the study period, while the HDR in Group 1 showed the lowest levels. From 1999 to 2007, the HDR significantly increased in all three county groups after implementation of the intervention, and correspondingly, the MMRs remarkably decreased. The HDR increased from 40.03% to 76.24% between 1999–2007 in Group 1, from 57.29% to 92.65% between 2001–2007 in Group 2, and from 77.01% to 92.56% between 2004–2007 in Group 3 (Table 2). The MMR in Group 1 declined from 129.47 per 100,000 live births in 1999 to 52.89 per 100,000 live births in 2007 with a reduction of 59.15% over the eight years; the MMR in Group 2 dropped from 90.58 per 100,000 live births in 2001 to 32.98 per 100,000 live births in 2007 with a reduction of 63.59% over the six years; the MMR in Group 3 decreased from 59.69 per 100,000 live births in 2004 to 31.94 per 100,000 live births in 2007 with a reduction of 46.49% over the three years. Group 3 had a higher reduction rate in the MMR in comparison with Group 2 and Group1 in the first three years of the intervention (Table 2). Over the study period, the AARR in the three county groups was 9.24% (95% CI: 8.61–9.89), 16.06% (95% CI: 13.08–18.93) and 18.61% (95% CI: 16.35–20.82), respectively (Figure 1).

**Table 2:** Trends of Maternal Mortality Ratio and Hospital Delivery Rate in the three priority county groups in China, 1999–2007.

	Live births	Maternal death		Hospital delivery	
		N	MMR	HDR (%)	RACS
**County Group 1**					
1999	1501484	1944	129.47	40.03	
2000	1532113	1856	121.14	42.91	−0.89
2001	1532286	1488	97.11	48.03	−1.40
2002	1471883	1385	94.10	50.68	−1.23
2003	1440507	1351	93.79	53.31	−1.04
2004	1427248	1202	84.22	58.29	−1.06
2005	1422350	1082	76.07	64.34	−1.03
2006	1455042	978	67.21	69.60	−1.09
2007	1501294	794	52.89	76.24	−1.26
**County Group 2**					
2001	183261	166	90.58	57.29	
2002	181829	151	83.05	63.02	−0.83
2003	183412	121	65.97	69.75	−1.42
2004	196099	142	72.41	76.18	−0.72
2005	197289	100	50.69	82.59	−1.41
2006	210165	68	32.36	86.26	−2.18
2007	218285	72	32.98	92.65	−1.86
**County Group 3**					
2004	2204880	1316	59.69	77.01	
2005	2351403	1092	46.44	81.68	−3.66
2006	2426127	952	39.24	88.12	−2.71
2007	2554429	816	31.94	92.56	−2.98

N: Number of maternal deaths.

MMR: Maternal Mortality Ratio (per 10,000 live births).

HDR: Hospital Delivery Rate.

RACS: Ratio of Average Change Speed.

The RACS differed in the three groups as shown in Table 2. From one year before to three years after the intervention, the RACS of the MMR was −1.23, −0.72 and −2.98 in Group 1, Group 2 and Group 3 respectively. During the first six years after the intervention, the RACS of the MMR was −1.03 and −1.86 in Group 1 and Group 2 respectively.

From 2004 to 2007, the ACS of the HDR was 6.32% and the ACS of the MMR was −18.81% in Group 3, and the RACS of MMR and HDR was −2.98. However, before the implementation of the intervention, the ACS of the HDR in Group 3 was 4.69% as HDR increased from 64.05% in 2000 to 77.01% in 2004, and the ACS of the MMR is −1.72% during the same period, as the MMR decreased from 64.03 per 100, 000 live births in 2000 to 59.69 per 100, 000 live births in 2004. Thus, during the period of 2000–2004, the RACS of MMR and HDR in Group 3 was −0.36. However, during the same period of 2000–2004, the intervention has been implemented in Group 1, the RACS in Group 1 was −1.09, when the ACS of the HDR was 7.96% and the ACS of the MMR was 8.69%. While from 2000 to 2007, the ACS of the HDR in Group 1 was 8.56% and the ACS of the MMR was −11.17%.

### The leading causes of maternal death in project counties

**Figure 2:**
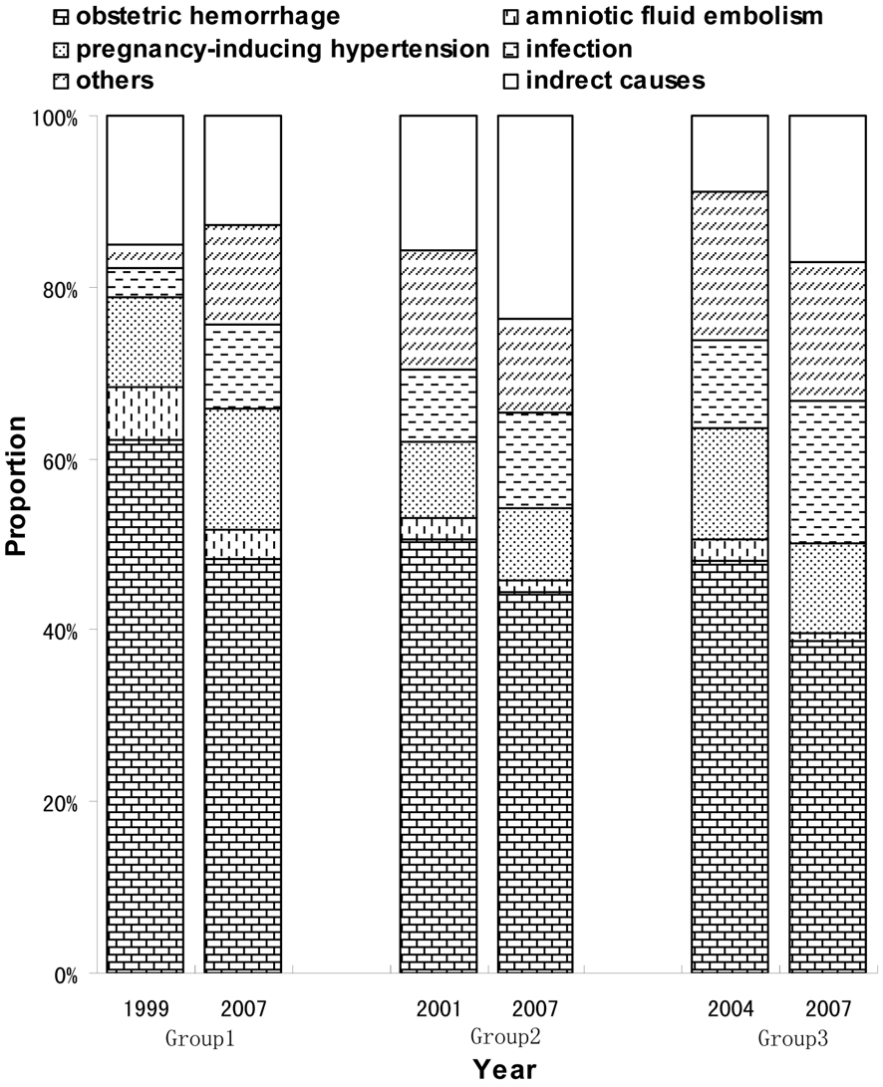
Distribution of causes of maternal deaths in three county groups during implementation of the intervention in China, 1999–2007.

During the study period, obstetric hemorrhage was identified as the most prevalent cause of maternal death, followed by pregnancy-induced hypertension and amniotic fluid embolism in the three county groups. The MMR due to obstetric hemorrhage compared to all causes in maternal death was the highest in Group 1 and the lowest in Group 3. For example, the 2007 MMRs of obstetric hemorrhage were 25.58, 14.66 and 12.37 per 100,000 live births in the three county groups, respectively. Following implementation of the intervention, the three leading causes of maternal deaths among all causes declined significantly in all three county groups over the study period. Occurrence of obstetric hemorrhage in maternal death among all causes dropped from 62.14% to 48.36% between 1999 and 2007, from 50.61% to 44.45% between 2001 and 2007, and from 48.10% to 38.73% between 2004 and 2007, respectively in the three groups (Figure 2). The MMR related to obstetric hemorrhage declined significantly by 68.20%, 68.02% and 56.91% in the three county groups, with an AARR of 11.25% (95% CI: 10.23–12.25), 18.03% (95% CI: 13.84–22.01) and 24.90% (95% CI: 21.65–28.02), respectively. Meanwhile, the MMR from pregnancy-induced hypertension also significantly declined in all three groups, but mortality from amniotic fluid embolism decreased only in Groups 1 and 3, but not in Group 2. Most noticeably, the MMR caused by infection in Group 1 increased during the study period between 1999 and 2007 (Table 3).

**Table 3:** MMR changes in the leading causes of maternal deaths by each priority county group in China, 1999–2007.

	Obstetric hemorrhage		Pregnancy-induced hypertension		Amniotic fluid embolism		Infection	
	N	MMR	N	MMR	N	MMR	N	MMR
**County Group 1**								
1999	1208	80.45	205	13.65	119	7.93	68	4.53
2000	1145	74.73	196	12.79	110	7.18	48	3.13
2001	888	57.95	166	10.83	77	5.03	41	2.68
2002	804	54.62	149	10.12	94	6.39	54	3.67
2003	797	55.33	130	9.02	69	4.79	58	4.03
2004	690	48.34	133	9.32	52	3.64	57	3.99
2005	623	43.8	129	9.07	29	2.04	61	4.29
2006	537	36.91	116	7.97	34	2.34	66	4.54
2007	384	25.58	112	7.46	26	1.73	78	5.20
Reduction rate (%)	−68.20		−45.36		−78.15		14.72	
AARR (%)	−11.25 (95%CI: −12.25–10.23)		−6.61 (95%CI: −8.95–4.20)		−17.80 (95%CI: −21.10–14.37)		8.12 (95%CI: 3.91–12.50)	
**County Group 2**								
2001	84	45.84	15	8.19	4	2.18	14	7.64
2002	75	41.25	19	10.45	4	2.20	14	7.70
2003	68	37.08	11	6.00	5	2.73	6	3.27
2004	74	37.74	19	9.69	1	0.51	13	6.63
2005	49	24.84	11	5.58	1	0.51	12	6.08
2006	27	12.85	8	3.81	4	1.90	8	3.81
2007	32	14.66	6	2.75	1	0.46	8	3.66
Reduction rate (%)	−68.02		−66.42		−79.01		−52.03	
AARR (%)	−18.03 (95%CI: −22.01–13.84)		−15.59 (95%CI: −24.06–6.19)		−17.76 (95%CI: −34.33-2.99)		−10.51 (95%CI: −20.08-0.23)	
**County Group 3**								
2004	633	28.71	171	7.76	33	1.50	134	6.08
2005	478	20.33	141	6.00	21	0.89	127	5.40
2006	373	15.37	128	5.28	15	0.62	131	5.40
2007	316	12.37	86	3.37	7	0.27	136	5.32
Reduction rate (%)	−56.91		−56.59		−81.69		−12.40	
AARR (%)	−24.90 (95%CI: −28.02–21.65)		−22.44 (95%CI: −28.26–16.15)		−40.42 (95%CI: −52.21–25.74)		−4.05 (95%CI: −10.96-3.74)	

N: Number of maternal deaths.

MMR: Maternal Mortality Ratio (per 10,000 live births).

## Discussion

We have reported a significant geographic inequality of MMRs in China by analyzing the data from the National Maternal Mortality Surveillance System (NMMSS). Our study found that the MMRs in western China were the highest. We found that about 86.1% of the maternal deaths were avoidable and that obstetric hemorrhage was the leading cause of maternal death. The lack of education and technology were probably the major causes of the poor medical services in western China [17], [22], [23]. These publications have contributed not only to an understanding the situation of maternal mortality in China, but also to the effectiveness of the ‘Reducing and Eliminating’ intervention by Chinese government. The ‘Reducing and Eliminating’ intervention has been implemented in 1,000 priority counties in mid-western China. However, the NMMSS included 176 counties that only covered 14 priority counties among them. The 14 priority counties could not represent the changes in MMR and the efficacy of the intervention implemented in all priority counties. Several previous papers have suggested that reduction of the MMR in western China was probably due to the intervention, but no direct evidence was mentioned [17], [19]. In this study, we have analysed the change in MMR and evaluated efficacy of the intervention based on the data collected from all 1,000 priority counties. It is very important for the government to estimate the efficacy of the intervention and make further modifications and also provide valuable experiences that other developing countries can draw upon to achieve the MDG5 target.

Similar to other countries, the MMR in China is notably unevenly distributed geographically. There is a higher MMR in rural and western areas than in urban and eastern areas due to the unbalanced development of the economy and inequality of healthcare services [17]. In China, 80% of healthcare resources were allocated to urban areas, especially the metropolis [24], [25]. As a result of limited healthcare resources and an underdeveloped transportation system, Chinese women in mid-western rural areas traditionally deliver at home [26]. In 2005, the HDR in remote areas was as low as 40% [27], [28]. By learning from the experiences from international organizations that had run healthcare programs in poverty stricken areas of China [29], [30], we found that encouraging hospital delivery was the most important intervention in “Reducing and Eliminating” to reducing the MMR in the priority areas. To impart knowledge of pregnancy and health,to eliminate traditional delivery, to develop healthy habits, and to increase hospital delivery, “Reducing and Eliminating” has launched healthcare education and extended poverty medical assistance to improve accessibility of healthcare services for pregnant woman in mid-western China. The largest percentage of the program's funds has been allocated to aid women who can not afford to deliver in a hospital. These funds have increased every year. During the study period of 2000–2005, 20% of the program funds were used to aid the poor. In 2006 and 2007, the percentage jumped to 81.3% and 90% respectively. The program subsidized a total of 2,283,787 of lower-income pregnant women for hospital delivery during the eight years of study (2000–2007). Overall, the relief rate increased from 3.82% in 2000 to 38.54% in 2007 [31]. Our study indicates that the HDR gradually increased in all three groups during the study period. This change led to an average HDR by 47.6% between 2001 and 2007 in these areas [32].

The “Reducing and Eliminating” program dramatically reduced maternal death in the 1,000 priority counties in mid-western China during the study period of 1999–2007. The MMR decreased by about 50% in all three county groups with an average reduction rate of 9.24%, 16.06%, 18.61% per year, respectively, in each of the three groups. These reduction rates completely met the MDG5 target of 5.5% [3] and were even higher than those in East Asia and Maldives who earned the highest records of the AARR worldwide between 1990 and 2005 [2]. In addition, between 2000 and 2005, the reduction rate in Western China first exceeded that of Eastern China [17]. The HDRs in the three county groups significantly increased during the study period, which was consistent with the main aim of the program to advocate for hospital delivery and promote HDR.

MMR is affected by a multitude of factors, including economy, education, healthcare and public transportation. However, our results have shown that increases in HDR are highly associated with reductions in MMR of the three county groups during the study period. The increases were most likely due to the implementation of the intervention. It would make sense that the HDR would increase in correspondence to the development of Chinese economy and living conditions, but the post-intervention HDR in Group 3 increased much faster (6.32% per year) than the pre-intervention HDR (4.69% per year). In addition, during the same period, the post-intervention HDR in Group 1 also increased much faster than the pre-intervention HDR in Group 3. The national average rural HDR published in the 2000–2007 Chinese annual healthcare report implies that the intervention contributed to the increase in HDR in the priority counties (4.51% vs. 8.56% per year) [33]. Most importantly, a rapid increase in HDR shown shortly after the implementation of the intervention in the priority counties can be mostly attributed to the intervention, especially due to the reduction and reimbursement of the hospital delivery cost. This cost was a heavy burden for the poor women and families and could be solved by the intervention during this short time, in contrast, the economy, culture, and transportation could not change rapidly.

Analysis of the RACS before and after implementation of the intervention has shown that the MMR was reduced with an increase in the HDR in the priority counties. From 2004 to 2007, the RACS and the decline speed for the MMR (−2.98 and 18.81% per year) after implementation of the intervention were higher than those (−0.36 and 1.72% per year) in Group 3 before implementation. In addition, from 2000 to 2004, the RACS and the decline speed for the MMR in Group 1 (−1.09 and 8.69% per year) after implementation of the intervention were higher than those (−0.36 and 1.72% per year) in Group 3 during the same period before implementation. Thus, the increase in RACS after implementation of the intervention in the priority groups has suggested that HDR reduced MMR more effectively. This might be attributed to the improvement of obstetric service quality in the medical centers by the intervention. In addition to encouraging women to utilize hospital delivery, the obstetric service quality and techniques in the local medical centers have been greatly improved through the use of basic and emergency obstetric equipment and medicine, the additional training of local obstetric doctors, and the development of obstetric emergency centers and express ‘green channel’ referral networks.

The differences in RACS among the three county groups have suggested the different efficacies of the HDRs in reducing the MMR. This is probably associated with the basic obstetric service quality offered which differed in the three groups before implementation of the intervention. Among the three groups, Group 3 had a better economic and healthcare system and basic obstetric services. Therefore it was more sensitive to the implementation of the intervention. The results of the study suggest that this remarkable achievement in the reduction of maternal death in Western China is primarily attributed to the “Reducing and Eliminating” program initiated by Chinese government since 2000. The Chinese's practice in reducing MMR may provide valuable experiences and give confidence to other developing countries with a high or very high burden of maternal deaths that are working to reach the MDG5 target by 2015.

Although all 1,000 priority counties were selected from poverty areas in mid-western China, there was a difference in the proportion of individuals living in poverty. According to the national poverty definition standards [34], there were 72.62% of the population in Group 1 living in poverty, higher than that in Groups 2 (53.41%) and 3 (39.03%). Correspondingly, Group 1 carried the highest MMR as well as the lowest AARR among the three study groups. Furthermore, the reduction rate of MMR in Group 1 during the eight years of study (1999–2007) was lower than that in Group 2 during the six years of study (2001–2007). Poverty may lead to women's lack of education, low capacity of health services utilization and high burden of maternal death in these priority counties. Over the first three years after implementation of the intervention, Group 3 achieved the greatest reduction rate of the MMR, followed by Group 2. In the last three years of the study period (2005–2007), Group 2 surpassed Group 1 in the reduction rate of the MMR. These results therefore suggest that economic development and available healthcare resources are fundamental factors affecting the MMR. Without an improvement in these fundamental factors, it is very difficult to achieve a rapid and successful intervention of maternal death in resource-poor areas. Our result is consistent with the fact that low efficacy intervention appears in some resources-lacking developing countries [2], [7], [8]. Therefore, developing the economy in resource-poor areas and countries is very important to eventually improve the ability and accessibility of healthcare services and to maintain the MMR at a low level.

Similar to other Asian areas, obstetric hemorrhage was also the primary cause of maternal death in mid-western China during the study period. Before the implementation of the intervention, obstetric hemorrhage among all causes of death in mid-western China was much higher than that of other Asian regions [35] due to a high percentage of traditional home deliveries [26]. Since 90% of the maternal deaths caused by obstetric hemorrhage could be avoided [36], the “Reducing and Eliminating” program aimed to encourage rural women to use hospital delivery instead of home delivery and to improve the quality and accessibility of obstetric services in the rural and remote areas. The “Reducing and Eliminating” program significantly reduced the occurrence of obstetric hemorrhage in maternal women in mid-western China. In this study, although obstetric hemorrhage was still the leading cause of maternal death during 1999–2007, its occurrence dropped from 62.14% to 48.36%, 50.61% to 44.45% and from 48.10% to 38.73%, respectively, in the three county groups. The MMR due to obstetric hemorrhage also decreased significantly with an AARR of 11.25%, 18.03%, and 24.90%, respectively. Both the reduction rate and the AARR of obstetric hemorrhage in maternal death are greater than those of overall MMR in all three groups. Therefore, our study revealed that the “Reducing and Eliminating” program was an effective intervention for the reduction of MMR in the priority counties through targeting mainly obstetric hemorrhage, the number one cause of maternal death. Noticeably, the MMR from infection climbed. This suggests that many efforts are still urgently needed to improve the quality of obstetric care by encouraging hospital delivery in rural areas with underdeveloped health care.

Our study certainly has its limitations. The decrease in the MMR in the priority counties was not totally attributed to the implementation of the government intervention, since the influences of economic, culture, transportation and healthcare development on the MMR could not be completely excluded. However, the 1,000 priority counties with the most underdeveloped economies and the highest MMRs around the country would have not obtained a significant reduction in maternal death and improvement of obstetric healthcare in the short time period without implementation of the intervention, The data collected before 1999 from the MCHRRS was not considerably reliable due to lack of high quality control [18]. Consequently, this study was not able to conduct MMR research in the participating counties chronologically. Moreover, the scope of this study was limited to the participating counties. Accordingly no discussions or comparisons were revealed from this study as to the MMR difference of participating verses nonparticipating counties with similar backgrounds.

However, after tracking the progress of the study objects, we can conclude that the “Reducing and Eliminating” program played an important role in the dramatic reduction of the nationwide MMR and brought China on track with the MDG5 target within eight years.
